# State of the Field: Differentiating Intellectual Disability From Autism Spectrum Disorder

**DOI:** 10.3389/fpsyt.2019.00526

**Published:** 2019-07-30

**Authors:** Audrey Thurm, Cristan Farmer, Emma Salzman, Catherine Lord, Somer Bishop

**Affiliations:** ^1^Neurodevelopmental and Behavioral Phenotyping Service, Office of the Clinical Director, National Institute of Mental Health, National Institutes of Health, Bethesda, MD, United States; ^2^UCSF Weill Institute for Neurosciences, University of California, San Francisco, San Francisco, CA, United States; ^3^Semel Institute of Neuroscience and Human Behavior, David Geffen School of Medicine at UCLA, Los Angeles, CA, United States

**Keywords:** differential diagnosis, developmental delay, intellectual disability, autism spectrum disorder, DSM-5

## Abstract

The topic of this special issue on secondary versus idiopathic autism allows for discussion of how different groups may come to manifest autism spectrum disorder (ASD) or ASD-like symptoms despite important etiological differences. A related issue is that, because many of the social communication deficits that define ASD represent a failure to acquire developmentally expected skills, these same deficits would be expected to occur to some extent in all individuals with intellectual disability (ID). Thus, regardless of etiology, ASD symptoms may appear across groups of individuals with vastly different profiles of underlying deficits and strengths. In this focused review, we consider the impact of ID on the diagnosis of ASD. We discuss behavioral distinctions between ID and ASD, in light of the diagnostic criterion mandating that ASD should *not* be diagnosed if symptoms are accounted for by ID or general developmental delay. We review the evolution of the autism diagnosis and ASD diagnostic tools to understand how this distinction has been conceptualized previously. We then consider ways that operationalized criteria may be beneficial for making the clinical distinction between ID with and without ASD. Finally, we consider the impact of the blurred diagnostic boundaries between ID and ASD on the study of secondary versus idiopathic ASD. Especially pertinent to this discussion are findings that a diagnosis of ID in the context of an ASD diagnosis may be one of the strongest indicators that an associated condition or specific etiological factor is present (i.e., secondary autism).

## Introduction

Whereas the rest of this special issue is devoted to specific factors that relate to secondary versus idiopathic autism spectrum disorder (ASD), in this focused review, we discuss a diagnostic concern that cuts across this discussion: When and how should ASD be diagnosed in the presence of intellectual disability (ID) of varying degrees? This discussion is relevant because of the high rate of ID in what is being termed “secondary” or non-idiopathic ASD, similar to what was previously described as “complex” ASD; see Miles et al. (1), where specific genetic etiologies are identified as contributing to the manifestation of ASD. In fact, genes associated with ASD are often the same genes that are associated with ID (2, 3), validating both the phenotypic and genotypic overlap between these conditions. In addition, the rate of genetic abnormality associated with ASD is significantly higher in the presence of comorbid ID (4, 5). Thus, when it comes to discussions of primary/idiopathic versus secondary autism, considering the role of ID in diagnosis and phenotyping is critical.

**Figure 1:**
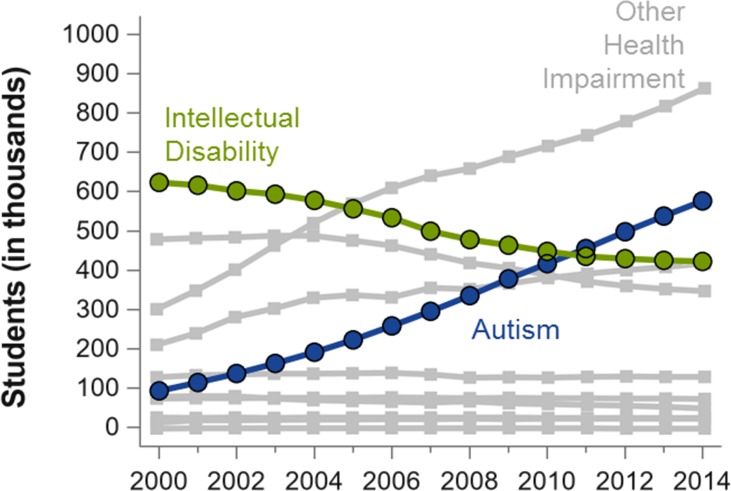
Number of students (in thousands) in the US who receive special education services pursuant to the Individuals with Disabilities Education Act, adapted from a previous publication (6). Numbers are plotted by the beginning of academic year (*X*-axis) and by diagnostic group, which are mutually exclusive. Other diagnoses not explicitly labeled include deaf-blindness, developmental delay, emotional disturbance, hearing impairment, multiple disabilities, and orthopedic impairment. The most common diagnoses, specific learning disabilities (in 2014, *n* = 2,278), and speech or language impairment (in 2014, *n* = 1,332) are not shown. Figure produced from data obtained from the U.S. Department of Education (7).

In this review, we focus on the impact of ID on the diagnosis of ASD. A few important trends underscore the importance of reviewing the *clinical* distinction between ID and ASD. One of these trends is the very large increase in ASD prevalence, accompanied by a strikingly similar decline in ID (6–8) (see **Figure 1**). The other related trend is the increase in research on genetic conditions, many of them previously considered to be disorders associated with ID (e.g., Fragile X Syndrome and Williams Syndrome), where increasingly high rates of ASD features and/or diagnoses are reported (6, 9). These trends lead to questions about how context and measurement may influence changes in diagnostic practice and also suggest inconsistencies in how individual clinicians and researchers distinguish ID from ASD.

Before proceeding, it is important to underscore the reasons for understanding how and when a distinction is made between ASD and ID, and when the two conditions are diagnosed simultaneously. As we approach the realization of treatments for the root cause of specific neurodevelopmental problems, such as preventative gene therapy (10), we must identify specific endpoints that will be used to evaluate their efficacy. The considerable etiologic and phenotypic overlap between ASD and ID makes the identification of relevant endpoints that are likely to be changed as a result of treatment (such as social communication abilities or IQ) difficult. More proximal to the everyday lives of individuals with these conditions, similar issues must be considered with respect to behavioral therapy goals and even educational categorization for classroom placements. Finally, from a theoretical perspective, exploration of the differences between diagnoses of ID and ASD begets questions about how to operationalize criteria for the ASD diagnosis itself. That is, the concept of *deficits* in social communication implies that these are unexpected or significantly more impairing than *delays* observed in the context of the individual’s other functional abilities. Whereas ID is associated with general deficits across developmental domains, ASD is in fact defined by the observation that social communication deficits *are particularly impairing*.

## Historical Context

Kanner’s first description of children with autistic disorder included delay in intellectual development as an associated feature in several of the cases described (11). Moving forward to formal recognition of autism as a disorder in the *Diagnostic and Statistical Manual of Mental Disorders, Third Edition* (DSM-III), criterion C specified gross deficits in language, and criterion D stipulated peculiar patterns of speech, if present (12). Thus, early descriptions of ASD assumed that a significant proportion of children would be minimally verbal, which overlaps considerably with ID (13). In fact, earlier epidemiological reports indicated that as many as 70% of individuals with ASD had co-occurring ID (14, 15). It was only recently that epidemiological reports reversed this trend, with some suggesting that ID was present in as few as 30% of children with ASD (6, 16). However, methdology may artificially deflate these rates (17). Recent studies have found that both minimally verbal and ID subsets of the ASD population are disporpotionately and increasingly underincluded in ASD treatment studies (18), neuroimaging studies (19), and ASD research in general (20).

The systematic exclusion of individuals with ID in ASD-focused research may affect the validity of current estimates for rates of ID in ASD (20). Further, it raises concerns about how clinicians are trained to diagnose ASD in the context of ID. Studies up to the early 2000s often focused on how to differentiate ASD from ID (without ASD), as this was a predominant referral question (21–24). However, recent literature on ASD screening and diagnosis has shifted to reflect the changing referral trends, focusing more on differential diagnosis among children with average IQ (e.g., differentiating between ASD and ADHD or ASD and language disorder) (25, 26). Studies that have continued to explore differentiation of ASD and ID are primarily focused on genetic syndromes (27–29), since ASD geneticists are looking to specific genetic conditions with increased rates of ASD for clues about how to derive treatments for ASD symptoms (30). In addition, ASD-focused research within rare genetic syndromes has been fueled to some extent by specialized funding resources targeting ASD.

## Diagnostic and Statistical Manual of Mental Disorders (DSM)

While diagnoses akin to ID were in earlier versions, beginning with DSM-III (12), the American Psychiatric Association introduced a multiaxial conceptualization. This system was maintained in DSM-IV, which placed mental retardation (the term previously used for ID) on Axis II. ID therefore served as a sort of “add on” that could be assigned in the presence of many other Axis I diagnoses, including ASD. This had an advantage of encouraging clinicians to consider each axis, and provided a clear structure for reporting different, though potentially associated, difficulties. However, this structure effectively invalidated a conceptualization of ID as a primary or autonomous mental disorder. While other organizations already had nosology for ID as a distinct condition (31, 32), the DSM-5 rid the multiaxial system so ID could be recognized as a primary diagnosis (33).

ID is now included as a freestanding diagnosis within the Neurodevelopmental Disorders section of the DSM-5. It may therefore be diagnosed alongside any other neurodevelopmental disorder (although cautions are given relating to diagnostic distinctions for several conditions, including ASD). The DSM-5 defines ID based on deficits in intellectual functioning *and* deficits in adaptive functioning. The option to assign ID as its own diagnosis, not in the presence of ASD or any other neurodevelopmental disorder, requires that clinicians have sufficient understanding of the clinical manifestation of ID. Given the shift in composition of ASD referrals to include a significant proportion of individuals with borderline range, average or above-average IQ, clinicians working with the “contemporary” ASD population may be far less experienced with ID (especially in the severe to profound range) than previous generations. It is worth noting that “With or Without Intellectual Impairment” is included as a specifier to be considered when assigning an ASD diagnosis under DSM-5; however, while this specifier can be made independently of an ID diagnosis, this specifier is redundant when an independent diagnosis of ID is made.

Specifiers for ID severity differentiate individuals based on ability to function independently across conceptual, social, and practical domains. The conceptualization of intellectual functioning as a generally normal distribution suggests that the majority of people with ID should be in the mild range, a supposition borne out by the data (34). Individuals with mild ID typically achieve verbal fluency and are often able to function with independence in at least some domains, whereas those with profound ID (representing a very small percentage of those diagnosed with ID) require 24-h care for all activities of daily living (35). Of particular relevance to the diagnosis of ASD in ID is the fact that social function is included in the conceptualization of ID severity, as it is expected that degrees of ID will be associated with increasing immaturity in social interactions. As described in the DSM-5, verbal or nonverbal modes of social communication may be observed, but the content will be limited. Expectations for social abilities decrease as severity increases; in or beyond the moderate range of ID, “individuals may not perceive or interpret social cues accurately” (p. 35) (33). Thus, as described below, clinicians are presented with the difficult task of determining when observed social deficits are attributable to an individual’s ID, and when an additional diagnosis of ASD is warranted.

At all levels of ID, the social communication deficits and interfering repetitive behaviors/restricted interests that confer a comorbid diagnosis of ASD result in even greater reductions in functional independence than if the individual only had a diagnosis of ID. This is reflected in studies showing that adaptive behavior skills are lower than expected based on IQ for individuals with ASD (36–39). Furthermore, data from clinical populations suggest that the distribution of ID severity among people with ASD is skewed downward, such that the rate of severe-to-profound ID is higher among those with ASD and ID than in those with ID alone (40). This is consistent with the detrimental effects of ASD on both attainment and measurement of cognitive and adaptive abilities (41, 42). With respect to the severity of ID, the fact that individuals with both ASD and ID are particularly likely to have a specific genetic etiology, and that severe to profound ID is more common in such cases of rare genetic syndromes than in the general ID population (43), results in a theoretical and non-hereditary “bump” in the normal distribution at the very low end of IQ (44).

## Diagnosing ASD in the Context of ID

Diagnosing mental conditions in individuals with ID presents challenges in general. For this reason, a separate diagnostic manual has been created to provide guidance about when DSM-5 criteria should be modified for the ID population (45). The Diagnostic Manual-Intellectual Disability-2 highlights complexities in distinguishing ASD in individuals with ID, but does not indicate any adaptations for the ASD diagnostic criteria beyond utilizing the DSM-5 requirement that deficits “exceed impairment consistent with the level of intellectual disability” (p. 131) (45). Similarly, Criterion E of the DSM-5 ASD criteria requires that “disturbances are not better explained by intellectual disability or global developmental delay” (p. 51) (33), though it does not specify how this should be determined. Thus, neither manual gives instructions about how or when ID may or may not “explain” symptoms of ASD. In addition, as discussed later, the ability to make such determinations may depend on the severity of ID and the age of the individual, as certain distinctions may be increasingly challenging at more severe levels of ID and/or at certain ages/developmental periods.

Research on specific genetic conditions associated with ASD has highlighted some of these diagnostic challenges, because ID is much more common when a genetic etiology is identified than in the idiopathic ASD population (46). There are examples from several genetic syndromes, including Fragile X Syndrome (47, 48), Dup15q Syndrome; (49), and Smith–Lemli–Opitz Syndrome (50), wherein lower IQ is associated with increased rates of ASD diagnosis. An exception may be when the ID is so severe that clinicians apparently find it impossible to identify *relative* deficits in social communication and play, given the extremely low mental age (51).

Variability in rates of ASD diagnosis within and across genetic syndromes may also depend on the clinician’s degree of reliance on standardized instruments versus clinical judgment (9). Moss and Howlin (28) provide a comprehensive review of the literature with respect to diagnosing ASD in individuals with genetic syndromes, urging even more caution when attempting to differentiate clinically relevant symptoms of ASD from ID. Results of studies that rely exclusively on scores or cutoffs from standardized measures without following manual-based instructions to carefully consider expert clinical judgment are likely to be especially misleading (52, 53). It is essential that clinicians use all available information about the individual to decide whether social communication skills that are below chronological age expectations are also lower than mental age/developmental expectations. This is challenging, especially when there are discrepancies in various aspects of mental age (e.g., verbal, nonverbal), when the individual’s mental age is lower than the minimum mental age assumed for the measure itself (27, 54), or when the clinical picture is complicated by comorbid sensory or other medical concerns. Therefore, depending on the study methodology and the clinical training and experience of the diagnosticians, rates of ASD diagnosis can vary tremendously between samples of individuals with the same syndrome.

## ASD Diagnostic Assessment of Individuals With ID

When considering if and how to make a diagnosis of ASD in an individual with ID, it is imperative that severity of ID be considered carefully. The mental age associated with ID in the severe to profound range of ID may not exceed 18 months, an age before which it may not be possible to assess certain abilities (e.g., spoken language, which is not expected to develop until 12 to 18 months of age). Thus, while there is no agreed-upon mental age that automatically triggers the exception described in the DSM-5 criterion E that “disturbances are not better explained by intellectual disability or global developmental delay” (33), diagnosticians need to keep in mind the possibility that severe ID might prevent valid assessment of certain ASD symptoms.

One convincing reason for not advising a minimum mental age requirement for the diagnosis of ASD is the need to diagnose children as young as possible. ASD is diagnosed as early as the second year of life (55), so requiring that toddlers meet a mental age threshold of 18 or 24 months (for example) could preclude an ASD diagnosis in very young toddlers and/or those with even slight cognitive delays (56). In addition, the symptoms of ASD may themselves deflate cognitive scores, creating circularity where young children with ASD cannot be diagnosed with ASD because their ASD has caused their test scores to be artificially low. The limited social responsiveness, interfering repetitive behaviors, and other behavior problems (e.g., hyperactivity, aggression, and inattention) common in children with ASD complicate cognitive testing in this group (57), potentially leading to lower scores (58). On the other hand, given longitudinal data showing that early (i.e., at or before age 3 years) nonverbal IQ scores below 70 usually persist into adulthood (41), clinicians need to be careful not to dismiss the prognostic significance of low IQ scores in young children with ASD.

Although diagnostic criteria do not explicitly state a developmental level under which a diagnosis of ASD is not advisable, ASD-focused research studies have begun to introduce specific thresholds for inclusion. The Simons Simplex Collection (SSC) required a mental age of 18 months for probands with ASD (59), and a large case–control cohort study of young children excluded children with mental ages under 24 months from analyses of the data (60). These decisions were based on research findings that widely used diagnostic instruments for ASD (i.e., the Autism Diagnostic Interview-Revised and the Autism Diagnostic Observation Schedule) are far less specific when used for individuals with nonverbal mental ages below 15 months (21), as well as recommendations from the test developers that the instruments be used cautiously (or not at all) with children with very low mental ages (61, 62). Such limitations in age and developmental level extend to other commonly used measures of ASD symptoms. The manual for the Social Responsiveness Scale states that all validation data were obtained from individuals with IQs above 70; therefore, “additional clinical judgment” is needed to interpret scores for individuals with ID (63). Both a thorough review of autism screening and diagnostic instruments (64) and a recent review of diagnostic instruments for preschoolers (65) suggested caution regarding how IQ can affect performance of these measures in general, with several examples of lower specificity in ID groups. For example, although limited sensitivity and specificity data for diagnostic measures such as the Diagnostic Interview for Social and Communication Disorders (DISCO) (66) and the Developmental, Dimensional and Diagnostic Interview (67) exist, two studies of the DISCO showed over-classification of individuals with low IQ (66, 68).

A common approach in research has been to proceed with an abundance of caution by restricting the participation of individuals with ID, although this reduces the generalizability of results. However, research on genetic syndromes cannot realistically employ mental age cutoffs for inclusion. They can employ minimum mental age cutoffs for individuals they deem capable of completing ASD diagnostic instruments, and apply even more stringent thresholds regarding whether scores from diagnostic instruments should be considered valid (28), but these practices have not been consistently adopted up to this point.

In addition to the complicating factor of cognitive impairments, individuals with ID associated with genetic conditions are likely to have physical disabilities. Sensory impairments affecting vision and hearing are among the most frequently identified, along with other neurologic conditions that include ataxia and epilepsy (69). The implications of this are twofold. First, these comorbidities confer further limitations to the standardization (and therefore validity) of ASD diagnostic instruments and are themselves often difficult to differentiate from symptoms of ASD. Second, individuals with such sensory impairments are at increased risk of ASD (70, 71). Interestingly, the DSM-5 states that it may be difficult to specify the severity of ID in individuals with sensory impairments due to limitations in administering and interpreting standardized measures in this population (33), but there is no consideration of sensory impairments in the differential diagnosis section pertaining to ASD criteria. As a result, there have been recent efforts to develop specific autism diagnostic measures that can be validated in samples of individuals with ID and specified sensory impairments (72).

### Assessment of Individuals With Mild to Moderate ID

Focusing on those individuals with ID who are in the mental age range appropriate for standardized ASD diagnostic instruments, one way of conceptualizing the diagnostic features of ID + ASD (versus ID alone) would be to separate basic social communication skills, which are relatively ASD-specific, from the more advanced social communication skills that may be difficult for individuals with a range of neurodevelopmental disorders, including ID (73). Analyses of instruments like the ADI-R and ADOS indicate that nonverbal communication behaviors that typically emerge in early development, such as eye contact, facial expressions, gestures, as well as the capacity to share enjoyment and participate in simple, back and forth games (e.g., peek-a-boo), are most specific to ASD (74, 75). This is consistent with older literature showing that, compared to children with ASD, children with ID show greater capacity for joint attention behaviors, showing and directing attention, range of directed affect, as well as use of socially appropriate eye gaze to social communication purposes (76–78).

Although these deficits in basic social communication behaviors tend to be relatively more specific to ASD, their diagnostic utility may decrease over time, as individuals with and without ASD acquire more skills. Consequently, as individuals progress in age and developmental level, and achieve higher levels of language, ASD instruments will necessarily focus more on higher-level social communication skills (e.g., conversation, ability to modify behavior to match social expectations). This complicates assessment of social communication in individuals with ID with fluent language abilities; for many individuals with mild to moderate ID who do not have ASD, their language skills are at least commensurate with nonverbal ability, and it would not be uncommon for them to be capable of completing Module 3 or 4 of the ADOS, for example (79). However, ASD-focused assessment tools for individuals with fluent language were developed primarily with non-ID populations (61, 62, 80, 81), reflecting the fact that most individuals with ASD who are capable of producing complex sentences also have average or above average nonverbal IQ (82). This also highlights the problem of identifying appropriate comparison groups for ASD research, because the degree of language deficit (or nonverbal-verbal IQ discrepancy) that occurs in some individuals with ASD does not occur as commonly outside of ASD (83). Therefore, in addition to expanding standardization of tools such as the ADOS to capture individuals at older ages who are not able to verbally respond to modules traditionally designed for an older population (84), it will also be important to further develop our understanding of individuals with ID without ASD. Specifically, assessing large groups of individuals with ID without ASD will be critical to develop tools that can detect subtle differences in higher-level social communication skills even if the groups differ significantly in language and/or nonverbal cognitive skills.

### Severe to Profound ID

The data required to inform the differentiation of ASD from ID, especially in the severe to profound range, are limited. For individuals with severe or profound ID, the term profound intellectual and multiple disabilities (PIMD) is often used, to reflect the accompanying physical disabilities and/or sensory deficits (e.g., vision and/or hearing impairments) (85). Individuals with PIMD are the least common generally, but this group is overrepresented in many genetic conditions implicated in the etiology of ASD (46). Despite the interest in measuring ASD symptoms in this group, the fact remains that standardized measures of ASD symptoms are generally not appropriate for this purpose (21, 61, 62). ASD diagnostic measures were designed to identify individuals with ASD primarily, and therefore they assume profiles of ability that are more traditionally characteristic of ASD. For example, motor skills tend to be relatively preserved in ASD even among those with very low IQ and language abilities (86, 87), as are other basic sensory abilities such as vision and hearing. As a result, standardized diagnostic instruments like the ADI-R and ADOS were not validated for individuals with interfering vision, hearing, or mobility limitations or other significant motor impairments (e.g., ataxia, dystonia), which are common co-occurring problems for many individuals with PIMD, especially those with genetic syndromes and/or neurological conditions (e.g., epilepsy).

Restrictions placed on use of standardized diagnostic tools have had downstream effects on our ability to conceptualize ASD in the context of ID. As the use of these tools in research on ASD and neurodevelopmental disorders becomes more widespread, results are fed back to inform clinical and policy decisions, shaping diagnostic systems like DSM-5. Thus, an unintended consequence of the fact that these tools were not designed or validated for individuals with PIMD is that people with PIMD may not be included in ASD research (other than studies that focus on specific genetic conditions), and empirical data about which behaviors best differentiate ASD in the context of severe to profound ID are not available. The need to operationalize specific differences between ID with and without ASD is clearly apparent, but the data to do this are limited in part by who was able to undergo valid standardized assessment of social communication and repetitive behaviors using currently available tools. Fortunately, there have been recent attempts to actually create diagnostic tools that are specifically tailored and standardized in samples of individuals with ID that include severe-to-profound ID (88), as well as modifications of the ADOS that are more appropriate for minimally verbal and older individuals with a nonverbal mental age of at least 18 months (84).

## Application of Criterion E: Clinical Guidelines

Best practice clearly encourages clinical judgment over prescriptive algorithms for the differentiation of ASD and ID at various chronological and mental age levels. Nevertheless, the following guidelines may be helpful to consider:

a) When evaluating an individual with ID for a potential diagnosis of ASD, it is necessary to be aware of the child’s cognitive ability (based on IQ or developmental test scores) and to understand any sources or clinical manifestations other than cognitive ability that may have influenced those scores. Thus, the diagnosing clinician should either directly administer the measure of cognitive functioning or obtain a detailed account of the behaviors that contributed to the IQ scores. Along with measurements of adaptive behavior, this information will provide context not only for the individual’s developmental level, but also for the behavioral, motor, and/or sensory impairments that may be contributing to the overall presentation.
b) An assessment of potential motor and sensory impairments (e.g., vision, hearing) should be incorporated into the differential diagnosis, especially given the increased prevalence of these impairments in individuals with a neurogenetic condition and/or severe to profound ID. If sensory or motor impairments are present, it is important to consider tools that are validated in these particular populations, and also to consider using a team approach to diagnosis that includes experts in the relevant sensory and/or motor deficits.
c) Given that Criterion E states “to make comorbid diagnoses of ASD and ID, social communication should be below that expected for general developmental level,” the diagnostician must evaluate his/her ability to determine whether observed deficits are consistent with expectations for the individual’s developmental level.
  i. The clinician must consider the behaviors expected at a given developmental level. However, the chronological age of the child is also relevant, as the effect of life experiences on behaviors cannot be ignored. For example, expectations for an 18-month mental age differ in the context of a 4-year-old versus a teenager. As a result of more life experiences, the teenager may be able to sit for longer periods or time; have more expertise with certain devices, objects, or toys; and be more compliant with routines and simple daily living tasks.
  ii. In the case of young children, the DSM-5 warns that distinguishing ID from ASD may be particularly difficult. At very young ages, it may be difficult, or even impossible, to determine social communication “delay” from “deviance,” since uneven developmental profiles are more subtle when children are too young to have developed many skills.
  iii. Related to the above, it is important to distinguish between when a symptom is present, but criterion E is met (the disturbance is accounted for by global developmental delay or ID so it should be considered “not applicable”), including when it is not possible to determine whether a symptom is present. In other words, each symptom should be considered “present,” “absent,” or “not applicable.” For instance, if motor impairments such as ataxia or motor apraxia interfere with a child’s ability to make purposeful movements such as pointing or other gestures, a rating of “not applicable” may accurately capture the criterion of deficits in specific nonverbal communication behaviors. Similarly, as discussed previously with respect to minimum mental age requirements, it may not be possible to judge presence/absence of a symptom if an individual has not achieved certain developmental skills. In such cases, a child may be observed to “grow into” his/her ASD symptoms in later childhood, once he/she acquires the cognitive, language, and/or motor skills required to assess social communication and play. A simple example of this would include a child who lacks necessary motor or cognitive skills to play with toys in any capacity, but then begins exhibiting highly repetitive play once these skills develop.
d) In the case of older individuals, the developmental trajectory may be particularly useful. Specifically, the clinician should consider whether social communication delays have always been approximately commensurate with other domains of development, or whether social communication delays have at times been an isolated or more significant impairment. It is also important for clinicians to consider that ASD diagnostic status may change. For example, as noted above, it may not be possible to judge the presence of ASD in a child with very significant ID until he/she attains a certain mental age equivalent. On the other hand, in cases of mild to moderate ID, clinicians must be careful when assigning ASD diagnoses in later childhood, adolescence, or adulthood. It is unlikely for any individual (with or without ID) to suddenly manifest social communication deficits and repetitive behaviors beyond the early childhood period that warrant an ASD diagnosis. In cases when ASD is suddenly being considered in an older individual with ID, the clinician must carefully consider whether observed social communication difficulties (e.g., difficulty related to same-aged peers, minor conversational difficulties, mild social disinhibition) are simply a reflection of social immaturity attributable to their ID or potentially changes in mental state in reaction to life transitions (e.g., more limited social exposure post high school).
e) While the administration of autism screening and diagnostic tools may be helpful in a variety of situations, clinicians must first consider the consequences of scoring and interpreting the results. For both research and clinical purposes, interpretations of the data may be significantly limited by characteristics commonly observed in individuals with ID, which may affect scoring on autism symptom tools. Examples are ataxia, recent onset of independent walking, significant dysarthria or motor apraxia, and visual or hearing impairments. Thus, even if the clinician intends to rely upon qualitative behavioral observations, the use of a given tool in any capacity may still be problematic if the tool is not appropriate for an individual.
f) When applying clinical judgment, biases and other motivations must be considered. The use of a multidisciplinary team may be most helpful to allow for multiple observers and perspectives. For clinicians whose primary training is in ASD, it may be necessary to seek substantial consultation when an individual presents with certain medical problems, neurological issues, motor impairments, etc. that are less common in individuals with ASD without ID. Clinicians must recognize that standardized tools perform differently in different populations and that their own biases and/or idiosyncrasies in how they normally “weight” certain scores or observations will not apply in the same way to all clinical groups. Further, clinicians must have sufficient understanding of the individual’s social history, including factors relating to family, socio-cultural, and service-related needs that may be important in teasing out caregiver’s responses to questions about the individual’s ability to respond to different social situations.

## Research Implications

There are important research implications for the preceding clinical discussion. The differentiation between ID and ASD is perhaps especially important in the research setting, where studies of genetic conditions associated with ID and ASD are proliferating (89). In order to decide between ASD features versus ID as a primary outcome of a trial, for example, the research team must be able to adequately assess both and understand which is more prominent. Further, this discussion highlights the importance of systematic inclusion of individuals with all levels of intellectual ability in the development and validation of instruments, diagnostic or otherwise, for measuring ASD symptoms. As mentioned earlier, some studies may choose to use measures even if they are not validated for ID, but for research purposes only. Even if this tiered approach is employed, some members of multidisciplinary teams may not be privy to the important issues that they must consider when interpreting scores from instruments not validated in individuals with ID. Thus, it is essential that a knowledgeable member of the study team is involved in any analysis or report that includes such phenotypic findings. Finally, the difficulty of differentiating ASD and ID highlights the need for further research on the operationalization of DSM-5 ASD criteria (and other classification systems) in the context of moderate-to-profound ID.

## Conclusion

As diagnostic tools for ASD are distributed and used more widely, and as awareness (and prevalence) of ASD continues to rise, researchers and clinicians are increasingly faced with extremely complex referrals for ASD diagnostic assessment. Indeed, the complicating presence of ID has been recognized since diagnosis of autism was first proposed. Further, ID is as heterogeneous as ASD, so strategies that may be helpful in distinguishing ASD from ID in individuals of a certain age and/or level of severity may not be applicable to others. Nevertheless, the specific considerations outlined above may be helpful to enhance both research and practice when evaluating and treating children with neurodevelopmental disorders.

Although the focus of this work was to help clarify the distinction between ID and ID + ASD, we must also emphasize the commonalities of the two conditions, which indicate that many interventions may be beneficial regardless of which neurodevelopmental disorder is diagnosed. Early intervention services (90) and services focused on improving communication (91) are indicated for children with any significant developmental delay and should be initiated at the time this concern is identified, rather than waiting for a diagnosis of ASD or ID to be made (92). Although, depending on locality, service provisions may differ considerably between individuals diagnosed with ASD versus ID, the perception is that individuals with ASD are generally afforded more comprehensive services (93, 94). This has motivated many parents to seek out an additional ASD diagnosis in order to secure additional services and has further muddled the question of when and for whom a comorbid ASD diagnosis is appropriate. To advance our understanding of the diagnostic distinctions between ID with and without ASD, there is an urgent need for changes in service provision that emphasize individual needs over diagnostic classification.

## Data Availability

The datasets for this manuscript are not publicly available because consents allow for limited data sharing. Requests to access the datasets should be directed to Somer Bishop, Somer.Bishop@ucsf.edu.

## Author Contributions

AT and SB initially drafted this manuscript, and CL, CF, and ES provided extensive editing and specific contributions.

## Funding

This work was partially supported by the Intramural Research Program of the NIMH (1ZICMH002961) to AT and CF, as well as R01HD093012 to SB.

## Disclaimer

The content is solely the responsibility of the authors and does not necessarily represent the official views of the National Institutes of Health.

## Conflict of Interest Statement

CL and SB receive royalties from Western Psychological Services for sales of ASD diagnostic instruments [CL for the Autism Diagnostic Interview-Revised, the Autism Diagnostic Observation Schedule (ADOS), and the ADOS-2; SB for the ADOS-2].

The remaining authors declare that the research was conducted in the absence of any commercial or financial relationships that could be construed as a potential conflict of interest.
